# The Influence of Parental Educational Attainment on the Partnership Context at First Birth in 16 Western Societies

**DOI:** 10.1007/s10680-017-9421-9

**Published:** 2017-04-26

**Authors:** Judith C. Koops, Aart C. Liefbroer, Anne H. Gauthier

**Affiliations:** 1 Netherlands Interdisciplinary Demographic Institute (NIDI/KNAW), Lange Houtstraat 19, 2511 CV The Hague, The Netherlands; 2 University of Groningen, Groningen, The Netherlands; 3 Department of Epidemiology, University Medical Centre Groningen, University of Groningen, Groningen, The Netherlands; 4 Department of Sociology, Vrije Universiteit, Amsterdam, The Netherlands; 5 Faculty of Behavioural and Social Sciences, University of Groningen, Groningen, The Netherlands

**Keywords:** Fertility, Childbearing, Cohabitation, Single parenthood, Europe, North America

## Abstract

In the US, growing up with parents with a low socio-economic status (SES) has been shown to increase the chance of having a birth outside marriage. However, less is known about the influence of parental SES in other Western countries. The current paper examines the association between parental educational attainment with the partnership context at first birth in 16 European and North American countries, by differentiating births within marriage, within cohabitation, or while being single. Moreover, we test whether the association between parental education and partnership context at childbirth changes over cohorts and whether its influence changes when controlling for own educational attainment. Data from the Generations and Gender Programme were used, as well as data from the American National Survey of Family Growth, the Canadian General Social Survey, and the Dutch Survey on Family Formation. The results show that in North American and East European countries, but not in West European countries, lower parental education increases the risk of having a birth within cohabitation. Moreover, in North American countries and half of the West and East European countries, lower parental education increases the risk of having a birth while being single. The association of parental education with the partnership context at birth tends to change furthermore over cohorts, although no clear pattern could be observed between countries. The study suggests that the intergenerational transmission of education is an important mechanism in explaining the influence of parental education, although other mechanisms also appear to be at work.

## Introduction

Over the past decades, the percentage of children born outside marriage (non-marital births) in Western societies has increased strongly. Within the European Union, the percentage of all births to unmarried women increased from 9 per cent in 1980 to 40 per cent in 2011 (Eurostat 2006, 2015), and in the US it increased from 18 per cent in 1980 to 41 per cent in 2013 (Martin et al. 2015). ‘Non-marital births’ is an umbrella term including births to cohabiting couples and to persons without a coresidential partner. The increase in non-marital births is mostly due to an increase in births within the context of cohabitation (Kennedy and Bumpass 2008; Kiernan 2004). As a consequence, births to cohabiting parents nowadays make up the lion’s share of the non-marital births in Europe. An increase in births to cohabiting couples is observed in the US as well, although births to single mothers continue to represent a significant portion of non-marital births (Heuveline et al. 2003).

In the US, growing up with parents with a low socio-economic status (SES) has been shown to increase the chance of having a birth outside marriage (Aassve 2003; Amato et al. 2008; Wu 1996). As such, the influence of parental SES on young adults’ partnership context at birth has been argued to be part of the reproduction of social inequality. Although this has been well documented in the US, much less is known about the influence of parental SES on partnership context at birth in other Western societies. In particular, it could be that in more egalitarian European countries, parental SES has less or no impact on partnership context at first birth. Moreover, while most US-based research has assessed the influence of parental SES on the chance of having a birth within or outside of marriage, it is likely that parental SES is differently related to the chance of having a birth within cohabitation and while being single.

Some cross-national studies have looked at the influence of own education on the partnership context at birth (e.g. Perelli-Harris et al. 2010b). However, focusing on parental education has the advantage of giving insight into the impact of socio-economic status across generations. Moreover, parental education is a measure that does not suffer from reverse causation, whereas own education is believed to do so in some contexts (Hoem and Kreyenfeld 2006). In the US, for example, births to single mothers are often teenage births which increase the chance of these women to drop out of school, thereby reducing their overall attained educational level (Hoffman et al. 1993).

In this paper, we study the influence of parental SES on the partnership context at first birth by examining how parental educational attainment influences whether first births occur within marriage, within cohabitation, or outside a coresidential partnership (i.e. single). We study union status at first birth, because starting a family is an important event in people’s lives which often triggers changes in partnership relations (Perelli-Harris et al. 2012).

We analyse data from 16 industrialized countries to test the hypothesis of the reproduction of social inequality in a variety of contexts. More specifically, we seek to answer three questions. First, what is the influence of parental educational attainment on the partnership context at first birth and how consistent is this influence across countries? Second, how consistent is this influence across cohorts within these countries? And last, to what extent does the association between parental education and partnership context at first birth change when a person’s own educational attainment is included? Analyses are carried out separately by gender to establish if similar patterns are found for men and women.

To answer our research questions, retrospective event history data are used from 13 European countries from the Generations and Gender Survey, as well as data from the American National Survey of Family Growth, the Canadian General Social Survey, and the Dutch Survey on Family Formation.

## Background and Previous Research

In this section, we discuss the literature that sheds light on the relationship between parental educational attainment and their children’s partnership status at birth. Given that parental education is a key dimension of parental SES, we will discuss not only studies on parental education, but also studies that focus on other aspects of parental SES.

### Influence of Parental SES on Marital Versus Non-Marital Births

In the US, low parental SES is associated with a higher risk of childbearing outside marriage. Research found that a lower and decreasing parental income increases the chance of experiencing non-marital childbirth for women (Aassve 2003; Högnäs and Carlson 2012; Wu 1996).

Various mechanisms have been suggested to explain this association. Parental SES is for example expected to influence the intergenerational transmission of norms and values regarding parenthood behaviour. In the US, parents with a lower SES are more likely to have experienced single parenthood, cohabitation, and separation (McLanahan 2009; Musick and Mare 2004). Studies have found that these parenthood behaviours are subsequently transmitted from parents to their children (Barber 2000; Högnäs and Carlson 2012; Liefbroer and Elzinga 2012; Musick 2002). This can be explained by socialization, as parents who themselves have not raised their children within marriage, might express more positive attitudes towards non-marital childbearing (Axinn and Thornton 1993; Thomson et al. 1992; Wu 1996). Alternatively, children might use their parents or their childhood family as a role model for their own behaviour (Forste and Jarvis 2007; McLanahan and Sandefur 1994; Thornton and Camburn 1987).

Another mechanism focuses on the marriage market of children with low SES parents. Children with lower SES parents have been argued to be more likely to have a lower income, be unemployed, or face other types of problems which make them less attractive on the marriage market (Oppenheimer et al. 1997). Information on parental resources may also be used by peers to assess the ‘quality’ or ‘potential’ of a partner, thereby influencing their decision to marry this person (Aassve 2003). Moreover, children of lower status parents are more likely to grow up in poor neighbourhoods with less attractive marriage partners (Edin 2000; Wu 1996). As having a child outside marriage carries little stigma among lower status groups in the US, people with low SES parents are expected to be less inclined to marry in response to a pregnancy, thereby increasing their likelihood of having a non-marital birth (Cherlin et al. 2008; Edin and Kefalas 2005).

Finally, lower SES parents invest less resources in their children than parents with a higher status, for example due to their lower income (Haveman and Wolfe 1995). Low status parents also tend to devote less (quality) time to their children (Bianchi et al. 2004). This is explained by the reduced ability of lower status parents to outsource household and care tasks, which leaves them with less time to spend on high-quality activities with their children (Baizán et al. 2014). Children with lower SES families are also more likely to experience stress during childhood, due to unemployment or separation of their parents, residential moves, etc. (Haveman and Wolfe 1995; Högnäs and Thomas 2016). The lower quantity and quality of parental investments and the increased likelihood of encountering stressful family situations could negatively influence the parent–child relationship and reduce parents’ ability to monitor and supervise their children (Axinn and Thornton 1993; Hofferth and Goldscheider 2010; Wu and Martinson 1993). As a result, children with lower SES parents are more likely to have early and unsafe sexual intercourse (Forrest 1994; Miller 2002), which increases their chance of childbearing outside marriage.

### Influence of Parental SES on Cohabiting Versus Single Births

The US literature on the impact of parental SES is traditionally focused on marital versus non-marital childbearing. Yet, given the increasing share of cohabiting parents, scholars have been stressing the need to distinguish three childbearing contexts: within marriage, within cohabitation, and outside a coresidential partnership (Amato et al. 2008; Hofferth and Goldscheider 2010). This is part of a broader discussion regarding the meaning of cohabitation, that is, whether it should be seen as an alternative to marriage, or instead as an alternative to singlehood (Bumpass and Raley 1995; Rindfuss and VandenHeuvel 1990).

In the US, cohabitation is mostly regarded as part of a pattern of disadvantage. Here, cohabitation is more common among the economic disadvantaged—for example because they have a lower chance to get married (e.g. Goldstein and Kenney 2001)—and is therefore viewed as a ‘poor man’s marriage’ or as an alternative to singlehood (Heuveline and Timberlake 2004; Kalmijn 2011; Oppenheimer 2003; Rindfuss and VandenHeuvel 1990). A similar pattern is observed in many Eastern European countries and other English-speaking countries (Heard 2011; Mikolai 2012; Perelli-Harris and Gerber 2011).

According to the Second Demographic Transition theory (SDT), improving living standards, weakening normative regulations, and increasing female autonomy resulted in an increasing need for self-development and individualism (van de Kaa 2001; Lesthaeghe 2010). Among other demographic changes, this manifested in an increased acceptance of cohabitation as an alternative to marriage and an acceptable context for childbearing (Kiernan 2001). This idea is underscored by the finding that a large group of cohabiting parents in Europe are in a committed relation often ending in marriage or view their union as an alternative to marriage—either because they ideologically reject marriage or because they believe marriage is irrelevant (Heuveline and Timberlake 2004; Hiekel and Castro-Martín 2014). A non-negligible fraction of cohabiting parents therefore seems to have consciously made the decision to (first) cohabit instead of getting married. Alternative explanations provided in the previous paragraph (e.g. the unavailability of suitable marriage partners, the lack of contraceptive use) might therefore be less relevant for this group. Thus, it is plausible that a low parental SES is less relevant for explaining births to cohabiting couples as compared to births to singles, which advocates a research design in which the three partnership contexts (marriage, cohabitation, and singlehood) are clearly distinguished from each other.

### Differences Across Societal Settings and Cohorts

US research concluded that children growing up with parents with a lower SES have a higher chance of having a child outside of marriage. A few studies in other countries came to the same conclusion. However, they were either performed in the UK where family formation patterns correspond highly to the US context (Berrington 2001; Ermisch 2001; Ermisch and Francesconi 2000; Rowlingson and McKay 2005), or quite long ago in a country—Sweden—where family formation patterns since changed drastically (Bernhardt and Hoem 1985). To our knowledge, no recent study investigates this link in several countries simultaneously, even though it is likely that the strength of this association depends on the societal context.

In the US context, children with lower SES parents are assumed to be more positive towards having a child without being married (Shattuck 2015). However, for births to cohabiting couples, this relationship is possibly different in societies that are more progressed in the Second Demographic Transition (see discussion above) or that legally treat cohabiting and married couples with children more equally (Perelli-Harris and Gassen 2012). Having a birth in cohabitation might thus be less related to low parental SES in West European countries. Moreover, low parental SES might be less related to having a birth while being single and within cohabitation in countries with more generous social policies (e.g. Nordic countries)—because children may have more opportunities to overcome their parents’ low SES as they grow up (Crettaz and Jacot 2014; OECD 2015). The same holds for countries where people from different SES groups are equally likely to circumvent unwanted or unintended pregnancies, for example through general access to contraceptives and abortion (Finer and Zolna 2014; Levels et al. 2014). Thus, parental SES might have a very different effect on the likelihood to have a birth in a certain partnership context in Western European countries, than in other countries, such as the US.

Differences in the influence of parental SES may also be observed across cohorts within countries. One potential factor is the advancement over cohorts in the SDT and the related shift in attitudes and norms towards childbearing in cohabitation. The SDT assumes that the higher educated are often among the first to adopt new demographic behaviours, followed by the rest of society (Lesthaeghe and Surkyn 1988). Recent changes in the legal system in some countries, such as France and Belgium, whereby cohabiting couples with children enjoy a similar legal treatment as married couples (Barlow and Probert 2004), may have led to smaller differences by parental SES. More general societal changes such as the increase in economic inequality in some countries (OECD 2011) may affect the influence of parental SES as well, for example, by increasing the difficulties that children with low status parents face when growing up.

### Potential Change in the Influence of Parental Education by Introducing Own Education

Previous research in the US has shown that the association of parental SES with partnership context often decreases when including own education in the model (Aassve 2003; Amato et al. 2008). Using panel data, Musick and Mare (2006) showed that in the US the poverty of the mother significantly increases the poverty risk of her daughter which is in turn correlated with the daughters’ family structure. In other Western societies, parental and children’s socio-economic status are often positively related to each other as well (Breen and Jonsson 2005). Therefore, the intergenerational transmission of SES could potentially explain the influence of parental education on partnership status at birth.

### Gender

Most research that study factors influencing the partnership context at birth has only considered women. Some research, focusing on the intergenerational transmission of living arrangements, found that the mechanism of socialization and role modelling is stronger for daughters than for sons (Axinn and Thornton 1993). It is assumed that parents put more effort in transferring their norms and attitudes related to family formation to their daughters than to their sons, since daughters are more involved in childrearing and therefore play a more important role in socializing the (grand)children (Raffaelli and Ontai 2004). Another study indicated that, while the intergenerational transmission of norms and attitudes was similar among sons and daughters, parents tried to transmit more traditional family norms to their daughters and more liberal norms to their sons (Barber 2000). These findings suggest that the influence of parental education on the partnership context at birth could differ between men and women. For example, the negative association between parental SES and the chance to have a birth outside of marriage could be stronger for daughters than for sons, because parents might transfer their norms regarding family formation more strongly to their daughters than to their sons, and because higher status parents might be more liberal regarding their sons’ behaviour than that of their daughters. However, we expect these differences to be small, since we have little reason to assume that other mechanisms mentioned in the theory section are gendered.

## Data and Method

### Data

To answer the research questions, information on 16 countries was analysed. We used the Generations and Gender Survey harmonized version 4.2—GGS (Fokkema et al. 2016) for the analysis of 13 countries. For Canada and the Netherlands, we used original datasets, respectively, the General Social Survey cycle 20—GSS (Béchard and Marchand 2008) and the Onderzoek Gezinsvorming (English translation: ‘Survey on Family Formation’)—OG (CBS 2012). For the US, we used the dataset of the National Survey of Family Growth—NSFG—which was harmonized by the Non-Marital Childbearing Network (Perelli-Harris et al. 2010a). Information on the year of collection, age range, and sample size is given in Table 1.

**Table 1:** Information of the datasets including: the name of the dataset, year of collection (collected), the age range (age) of the respondents, and the sample size

Country		Dataset	Collected	Age	Sample size		
					Total	**Women** ^a^	**Men** ^a^
*North American countries*							
Canada	CAN	GSS	2006	15–79	23,608	10,940	8595
US	US	NSFG	2006–08	15–45	13,495	7211	6046
*West European countries*							
Austria	AUT	GGS	2008–09	18–46	5000	2946	1982
Belgium	BEL	GGS	2008–10	18–82	7163	3355	3159
France	FRA	GGS	2005	18–79	10,079	5122	3969
Netherlands	NET	OG	2008	18–63	7811	3358	3033
Norway	NOR	GGS	2007–08	19–81	14,881	6847	6704
*East European countries*							
Bulgaria	BUL	GGS	2004	17–85	12,858	6407	5408
Czech Republic	CZE	GGS	2004–06	18–79	10,006	4470	4211
Estonia	EST	GGS	2004–05	21–81	7855	4994	2806
Georgia	GEO	GGS	2006	18–80	10,000	5259	4183
Hungary	HUN	GGS	2004–05	21–79	13,540	7025	5597
Lithuania	LIT	GGS	2006	17–80	10,036	4380	4338
Poland	POL	GGS	2010–11	18–84	19,987	10,717	7833
Romania	ROM	GGS	2005	18–80	11,986	5744	5741
Russia	RUS	GGS	2004	17–81	11,261	5759	3360

^a^ Sample size after deleting respondents with values missing on the (in)dependent variable(s)

The first wave of the GGS contains information on European and non-European Western societies. Key advantages of the GGS are that it includes information on the socio-economic status of parents and detailed (monthly) retrospective information on the occurrence and timing of cohabiting and marital partnerships, as well as fertility. For this paper, we were able to use data of four West and nine East European countries. Insufficient information on fertility history, partnership history, or parental educational attainment was available for Australia, Italy, Japan, and the Netherlands. Investigation of retrospective data of various countries of the GGS has suggested that there are issues with the quality of the fertility history of the German dataset (Kreyenfeld et al. 2010; Vergauwen et al. 2015). Germany was therefore dropped from the analyses. Data for Sweden were not yet available at the time of the data analyses.

Combining all datasets left us with information on 189,566 respondents. Due to values missing on the dependent and independent variables, information on 171,499 respondents was available for analysis.^1^

### Data Quality

Previous research has shown that men’s fertility reports are generally less reliable than women’s (Joyner et al. 2012; Rendall et al. 1999). Apart from being less accurate in providing dates, men tend to underreport the number of non-marital children. These data quality issues are especially acute for fathers who do not share a household with their children and who do not have a good relationship with the mother of the children (Joyner et al. 2012). This suggests that no great data quality issues should arise for men who had their first child within cohabitation or within marriage. However, it is possible that men who had their first birth while being single are less accurate in providing birth dates or might not report these births. Research has shown that underreporting can affect the influence of background variables on fertility outcomes, by declining the magnitude of the coefficients (Joyner et al. 2012). The strategy in this paper therefore involves to first assess the descriptive data of men and women, to check whether the information on single births of men appears to be reliable, before comparing and interpreting the results of the analyses.

### Variables

Our focus is on union status at first birth. The datasets contain information on the months and years in which children are born and the start and ending of cohabitations and marriages. By combining the fertility and partnership histories, we calculated the respondents' age at childbirth and constructed the variable partnership context at birth. We differentiated between three possible events, having a first birth within marriage, within cohabitation,^2^ or while being single. Respondents who did not experience the birth of a biological child before the age of 45 or at the time of the interview were right censored.

The key independent variable *parental educational attainment* is measured by combining information on the educational attainment of the respondent’s father and mother. Specifically, we used the mean of father’s and mother’s education, or the education of only one of the parents if the information was not available for both.^3^^,^ 4 To facilitate testing whether the influence of parental education differed across cohorts, parental educational attainment was centred around the country mean. In the Dutch OG, respondents were asked about their parents’ educational attainment only if they did not live with their parents at the moment of the interview. This is not a large problem since households consisting of children, parents, and grandparents are uncommon in the Netherlands (Coleman and Garssen 2002). In the GGS and the American NSFG, parental education was measured with the International Standard Classification of Education (ISCED97), which is a categorical variable. The Canadian GSS and the Dutch OG used a country-specific list.

We recoded all educational attainment data into the International Standard Level of Education (ISLED) coding system, with scores ranging between 0 and 100. The ISLED-coding system was recently developed by Schröder and Ganzeboom (2014) and has two important advantages compared to ISCED. First, it allows us to include the educational variable as a continuous variable, which facilitates the interpretation of the influence of this variable on the outcome variable (especially when examining a possible changing influence of the variable over cohorts). Second, for some countries more detailed information was asked in the original questionnaire, which could not be captured by the 6 levels of ISCED. By requesting the raw data of countries, and converting this directly into ISLED, a richer variable could be created for some of the European countries. Schröder (2014) provided country-specific information on the translation to ISLED for all countries in our dataset, except Georgia, the US, and Canada. For these countries, instead, a general conversion scheme was used which is based on the correspondence between ISCED and ISLED in all countries of the European Social Survey (Schröder and Ganzeboom 2014).

*Respondent’s own educational attainment* is included as a time-varying covariate by using information on the highest educational level (in ISLED) attained by the respondent in combination with information on the year and month in which this highest level was reached, thereby assuming that respondents remained enrolled in school continuously after finishing primary education.^5^ The educational level is made to increase linearly from age 15 until the age at which the highest educational level has been attained, after which it remains constant. If respondents indicated that they were still enrolled in school at the moment of the interview, we assumed continuous schooling until the timing of the interview.^6^ In case information on the timing of reaching the highest educational level was missing, the median age at reaching ISLED level^7^ in that country was used to impute the missing value.

*Cohort* is a continuous variable reflecting the year of birth of the respondent. Only for the descriptive results showing the changes in partnership context at birth over different cohorts, a categorical variable was constructed. This variable differentiates people born before 1955, between 1955 and 1975, and after 1975. Because of the younger age range for the data of Austria and the US, and because for the US only data collected in 2006–2008 was used, these countries lack a cohort with people born before 1955. For the interaction model testing the difference in the association of parental education over cohorts, cohort was centred around the year 1960, because the SDT assumes that in many countries changes in family behaviour started from the 1960s onwards (Lesthaeghe and Surkyn 1988).

As control variables, *age*, *age* ^2^, and *age* ^3^ were included to correct for the nonlinear effect of age on the chance of having a first child. These are time-varying covariates referring to the respondent’s age in months at any moment between the age of 15 and the timing of entry into parenthood (or the timing of the interview if a respondent did not have a biological child at the time of the interview or before the age of 45).

### Analytical Approach

A discrete-time competing risk model is estimated to test the association of the independent variables with the risk of having a first birth within cohabitation or while being single as compared to having a birth within marriage. This model examines the monthly risk of a respondent to experience one of the events, starting from the age of 15. The model is competing because as soon as a person has experienced an event, he or she is no longer at risk of experiencing any of the other events. The results of the multinomial logistic regressions are reported in relative risk ratios. A relative risk ratio greater than one indicates that an increase in the independent variable increases the risk of becoming a cohabiting or single parent compared to becoming a married parent. A relative risk ratio smaller than one indicates that an increase in the independent variable decreases the risk of having a first child within cohabitation or while being single compared to having a first child within marriage. Since we are interested in how trends differ between countries and between gender, all analyses are run separately by country and gender.

## Results

In this section, we start with discussing the results of women. In subsections 4.5 and 4.6, the results of men are compared to those of women.

### Descriptive Statistics

Table 2 gives an overview of the distribution of first births across partnership contexts. These results show that in all countries the overall percentage of women who have their first birth within marriage is higher than the percentages having their first birth within a cohabitation or outside a coresidential partnership. However, the percentage having their child in marriage clearly decreases across cohorts. The descriptive results of the most recent cohort should be interpreted with caution though, as most people born after 1975 had not yet experienced a birth of a child at the moment of the survey. In some countries, early births are more often non-marital births. Part of the lower percentage of marital births in the most recent cohort might thus result from the overrepresentation of early births in this cohort. However, in North America, Western Europe, Estonia and Hungary, the change in marital births is already visible when comparing women born before 1955 and between 1955 and 1975. As the larger part of these cohorts already experienced childbirth, it is very likely that the differences in these cohorts will remain. The decrease in the percentage of births within marriage is accompanied by an increase in the percentage of first births to cohabiting women. In most East European countries, with the exception of Estonia and Georgia, the percentage of births within cohabitation is lower than in West European countries. The pattern in the North American countries is slightly distinct from that in Europe. In the US and Canada, the diminishing percentage of first births within marriage in the younger cohorts coincides with an increase in both the percentage of births to cohabiting and to single women. In contrast, the percentage of first births to single women among European countries is relatively stable across cohorts.

**Table 2:** Weighted percentage of respondents having a first child within marriage, cohabitation, and while being single per country, cohort, and gender

Country	Cohort	Women			Men		
		Married	Cohabiting	Single	Married	Cohabiting	Single
*North American countries*							
CAN	1927–1955	90.9	2.1	7.0	89.5	2.3	8.3
	1955–1975	73.9	14.0	12.1	75.0	15.2	9.8
	1975–1991	43.4	35.0	21.7	42.5	36.3	21.1
	Total	77.4	11.5	11.1	78.5	11.5	10.1
US	1961–1975	67.3	13.6	19.1	66.1	16.2	17.7
	1975–1993	38.5	30.8	30.6	39.3	31.4	29.3
	Total	56.1	20.3	23.6	57.1	21.3	21.6
*West European countries*							
AUT	1963–1975	60.2	28.2	11.6	58.3	30.5	11.3
	1975–1990	44.5	42.8	12.7	46.9	40.4	12.7
	Total	55.6	32.5	11.9	55.7	32.7	11.6
BEL	1928–1955	85.5	3.7	10.9	89.5	4.1	6.4
	1955–1975	76.9	14.1	9.0	80.3	14.3	5.4
	1975–1990	51.5	41.6	6.9	48.7	44.1	7.3
	Total	76.6	14.0	9.4	81.3	12.8	6.0
FRA	1926–1955	82.1	4.6	13.3	88.0	5.4	6.6
	1955–1975	61.8	30.8	7.4	58.9	35.9	5.2
	1975–1987	38.2	49.9	11.9	32.7	58.0	9.3
	Total	70.0	19.5	10.6	72.5	21.4	6.2
NET	1945–1955	94.7	1.8	3.6	96.8	2.2	1.0
	1955–1975	80.6	16.8	2.6	80.3	18.1	1.6
	1975–1990	60.0	35.3	4.7	52.0	44.3	3.7
	Total	81.6	15.3	3.1	82.8	15.6	1.6
NOR	1927–1955	84.3	4.0	11.7	83.7	6.8	9.6
	1955–1975	47.9	40.8	11.3	45.8	46.7	7.5
	1975–1988	24.0	64.5	11.5	24.0	67.7	8.4
	Total	61.7	26.8	11.5	61.3	30.2	8.5
*East European countries*							
BUL	1919–1955	86.3	4.4	9.3	89.4	5.3	5.3
	1955–1975	87.3	7.7	5.0	84.7	11.6	3.7
	1975–1987	63.6	29.1	7.3	54.4	42.8	2.8
	Total	84.6	8.1	7.3	85.3	10.3	4.5
CZE	1926–1955	84.4	2.8	12.8	88.1	2.4	9.5
	1955–1975	79.1	7.5	13.4	82.2	8.2	9.6
	1975–1987	64.5	17.0	18.6	59.6	28.1	12.3
	Total	79.9	6.5	13.7	83.4	6.9	9.7
EST	1924–1955	83.5	8.3	8.2	86.4	9.0	4.7
	1955–1975	67.8	21.1	11.2	67.9	28.0	4.1
	1975–1983	35.4	52.1	12.5	31.3	63.6	5.2
	Total	73.3	17.0	9.7	73.9	21.7	4.5
GEO	1926–1955	82.7	11.8	5.5	83.7	12.0	4.3
	1955–1975	78.7	17.2	4.1	71.3	24.7	4.0
	1975–1988	57.8	37.7	4.5	53.8	40.9	5.3
	Total	77.0	18.3	4.7	74.3	21.5	4.2
HUN	1926–1955	93.7	1.4	4.9	92.0	2.1	5.9
	1955–1975	84.9	8.0	7.1	82.9	10.9	6.3
	1975–1983	66.5	24.0	9.6	63.5	30.8	5.7
	Total	88.3	5.6	6.1	86.7	7.2	6.1
LIT	1926–1955	86.8	2.5	10.7	89.5	2.0	8.5
	1955–1975	85.1	4.6	10.3	88.8	4.5	6.7
	1975–1988	74.0	12.9	13.1	79.5	13.1	7.3
	Total	84.2	4.9	10.9	88.0	4.6	7.4
POL	1927–1955	89.3	2.4	8.4	92.2	1.8	6.0
	1955–1975	87.5	4.9	7.6	88.9	5.2	5.9
	1975–1993	74.5	14.0	11.5	76.9	15.7	7.4
	Total	84.9	6.3	8.8	87.4	6.4	6.3
ROM	1925–1955	86.9	3.7	9.4	90.9	3.3	5.8
	1955–1975	87.4	7.8	4.8	89.0	7.5	3.6
	1975–1987	80.0	14.9	5.1	79.2	19.3	1.5
	Total	86.2	7.0	6.8	89.0	6.7	4.4
RUS	1923–1955	81.3	8.1	10.6	85.5	7.4	7.1
	1955–1975	82.6	8.8	8.6	84.8	9.9	5.4
	1975–1987	69.5	17.1	13.5	77.7	17.3	5.1
	Total	80.5	9.5	10.1	84.4	9.5	6.1

### The Role of Parental Educational Attainment

To answer the first research question on the association of parental education and partnership context at birth, we estimated a model including parental educational attainment, cohort, and the control variables age, age^2^, and age^3^. The results of these models are presented in Table 3. Panel 1 shows the results for the comparison between having a birth within cohabitation and having a birth within marriage, and Panel 2 for the comparison between having a birth while being single and having a child within marriage.

**Table 3:** Results of the multinomial logistic regression showing the association of cohort and parental educational attainment (P_educ) with the risk of having a first birth within cohabitation (Panel 1) or while being single (Panel 2) compared to the risk of having a first birth within marriage^ a^

Country	Panel 1: cohabitation versus married				Panel 2: single versus married			
	Women		Men		Women		Men	
	Cohort	P_educ	Cohort	P_educ	Cohort	P_educ	Cohort	P_educ
*North American countries*								
CAN	1.125***	.978***	1.117***	.984***	1.047***	.990***	1.028***	.995
US	1.073***	.973***	1.056***	.969***	1.032***	.978***	.998	.979***
*West European countries*								
AUT	1.063***	1.000	1.045**	1.003	1.023	.985**	.991	.997
BEL	1.089***	1.003	1.092***	1.001	.997	.990	.999	.997
FRA	1.083***	.995	1.090***	.999	.990*	.981***	1.009	1.002
NET	1.154***	1.007*	1.145***	1.010**	1.022	.993	1.064**	.993
NOR	1.096***	.981***	1.092***	.984***	1.025***	.973***	1.019***	.978***
*East European countries*								
BUL	1.065***	.953***	1.070***	.938***	.992	.990	.994	.997
CZE	1.047***	.979**	1.070***	.985	1.012***	.986**	1.006	.988
EST	1.060***	.985***	1.071***	.986***	1.027***	.987**	1.003	1.007
GEO	1.040***	.985***	1.051***	.987***	.991	1.003	1.008	.991
HUN	1.093***	.976***	1.094***	.978***	1.021***	.986*	1.009	.986*
LIT	1.048***	.985*	1.067***	.997	1.003	.995	.991	.997
POL	1.051***	1.002	1.072***	.997	1.009**	.998	1.007	.996
ROM	1.048***	.957***	1.063***	.930***	.973***	.996	.975***	.993
RUS	1.023***	.991**	1.017**	.998	1.003	.991**	.985*	1.006

^a ^ Controlled for age, age^2^, and age^3^ (results not shown in the table)

* *p* < .05; ** *p* < .01; *** *p* < .001

Panel 1 shows that in the North American countries, most of the East European countries (except Poland), and in Norway, women with parents with a lower educational level have a higher risk to have a first birth within cohabitation compared to a birth within marriage, than women with higher educated parents. In the Netherlands, women with lower educated parents instead have a lower risk of having a birth within cohabitation, compared to women with higher educated parents. In Austria, Belgium, and Poland, parental education is positively associated and in France negatively associated with the outcome variable, but in these countries the associations do not reach statistical significance. In all countries, the risk to have a first birth within cohabitation rather than within marriage increases across cohorts.

Panel 2 of Model 1 shows that in all countries, except Georgia, parental education is negatively associated with the risk of having a birth while being single compared to a birth within marriage, indicating that overall women with lower educated parents have a higher risk of having a birth while being single. The coefficients reach statistical significance in the North American, half of the West European (Austria, France, and Norway), and half of the East European countries (Czech Republic, Estonia, Hungary, and Russia).^8^ The risk of having a first birth while being single rather than while being married decreases across cohorts in France and Romania, but increases in the US, Canada, Norway, and a number of East European countries (Czech Republic, Estonia, Hungary, and Poland).

### Cohort Differences in the Role of Parental Educational Attainment

In order to examine whether the association of parental educational attainment and union status at childbirth differs across cohorts (research question 2), we re-estimated the previous model and included an interaction term between parental education and cohort. The main effect of cohort represents the association of cohort for women with an average level of parental education. The main effect of parental education can be interpreted as the estimated association of parental education for women born in 1960. The results are presented in Table 4.

**Table 4:** Results of the multinomial logistic regression showing the association of cohort and parental educational attainment (P_educ) and their interaction, with the risk of having a first birth within cohabitation (Panel 1) or while being single (Panel 2) compared to the risk of having a first birth within marriage ^a^

Country	Panel 1: cohabitation versus married						Panel 2: single versus married					
	Women			Men			Women			Men		
	Cohort	P_educ	P_educ* cohort	Cohort	P_educ	P_educ* cohort	Cohort	P_educ	P_educ* cohort	Cohort	P_educ	P_educ*cohort
*North American countries*												
CAN	1.121***	.987***	.999***	1.115***	.988**	.999*	1.045***	.992**	.999**	1.028***	.995	1.000
US	1.071***	.967***	1.001	1.047***	.981*	.999	1.029***	.976***	1.000	.995	.977**	1.000
*West European countries*												
AUT	1.069***	.982**	1.002***	1.048**	.998	1.001	1.029*	.961***	1.002**	.989	.999	.999
BEL	1.090***	1.011*	.999	1.092***	1.005	.999	.999	.989	1.001	.999	.997	.999
FRA	1.083***	1.003	.999**	1.091***	1.006	.999	.989*	.980**	.999	1.009	1.002	.999
NET	1.157***	1.016**	.999*	1.150***	1.023***	.998***	1.023	.992	1.000	1.066**	.988	1.001
NOR	1.092***	.992**	.999***	1.089***	.990***	.999***	1.023***	.975***	.999	1.016***	.978***	.999
*East European countries*												
BUL	1.068***	.951***	1.001	1.052***	.954***	.999**	.990*	.990*	.999	.989*	.992	.999*
CZE	1.047***	.986	1.000	1.069***	.991	1.000	1.011**	.988**	.999	1.005	.990	1.000
EST	1.061***	.993	1.001***	1.070***	.994	1.000	1.026***	.992	1.000	1.004	1.008	1.000
GEO	1.044***	.986***	1.001***	1.058***	.988***	1.001***	.992	1.006	1.000	1.011	.993	1.001
HUN	1.088***	.990	.999	1.089***	.992	.999**	1.016***	.988*	.999	1.004	.982*	.999*
LIT	1.049***	.986	1.000	1.065***	1.007	.999	1.006	.994	1.000	.988*	.996	.999*
POL	1.051***	1.001	1.000	1.073***	.997	1.000	1.010**	.997	1.001***	1.007	.996	.999
ROM	1.046***	.962**	1.000	1.051***	.943***	.998**	.972***	.991	.999	.973***	.988	.999
RUS	1.026***	.990**	1.001**	1.021**	.996	1.001*	1.006	.991**	1.001**	.986*	1.006	1.000

^a ^ Controlled for age, age^2^, and age^3^ (results not shown in the table)

* *p* < .05; ** *p* < .01; *** *p* < .001

The results of the main effect of parental education on the risk to have a birth in cohabitation (Panel 1) and the interaction effect with cohort show that in Canada and Norway the negative association of parental education is more negative for younger cohorts. In Austria, Estonia, Georgia, and Russia, the negative association instead becomes less negative with consecutive cohorts (the same is found in the US and Bulgaria, but the associations do not reach significance). The opposite is found in the Netherlands, in this country the positive association becomes less positive with consecutive cohorts (in Belgium this association does not reach significance). In France, the positive association for older cohorts changes into a negative association for more recent cohorts.

For the model comparing the risk of having a birth while being single versus within marriage (Panel 2), only for a few countries significant interaction effects are found. In Canada, the negative association of parental education is more negative for consecutive cohorts, while in Austria and Russia the negative association become less negative. In Poland, the negative association for older cohorts changes into a positive association for younger cohorts.

### Change in the Role of Parental Education by Introducing Own Education

To examine to what extent the association of parental education with the partnership context at birth changes by including own educational attainment, a model was estimated including both variables.^9^

When comparing Panel 1 of Tables 3 and 5, we observe that in all North American and East European countries (except Poland) the negative coefficient of parental education and the risk to have a first birth within cohabitation versus within marriage becomes less negative when own educational attainment is included in the model. However, in most countries (Canada, the US, Bulgaria, Estonia, Georgia, and Romania), the negative association remains statistically significant. In Norway, the initial negative association declines slightly, but remains significant too, while in the Netherland a marginal reduction in the size of the positive coefficient reduces the initial significant association to non-significance. In Poland, Austria, Belgium, and France, the coefficients become slightly more positive or less negative, but remained non-significant.

**Table 5:** Results of the multinomial logistic regression showing the association of cohort, own educational attainment (O_educ), and parental educational attainment (P_educ) with the risk of having a first birth within cohabitation (Panel 1) or while being single (Panel 2) compared to the risk of having a first birth within marriage^a^

Country	Panel 1: cohabitation versus married						Panel 2: single versus married					
	Women			Men			Women			Men		
	Cohort	O_educ	P_educ	Cohort	O_educ	P_educ	Cohort	O_educ	P_educ	Cohort	O_educ	P_educ
*North American countries*												
CAN	1.133***	.972***	.983***	1.121***	.976***	.990**	1.056***	.956***	.997	1.033***	.971***	1.003
US	1.068***	.956***	.986***	1.049***	.951***	.987*	1.028***	.964***	.986***	.993	.964***	.990*
*West European countries*												
AUT	1.064***	.997	1.001	1.045**	1.004	1.003	1.024	.987**	.989*	.991	.984*	1.000
BEL	1.090***	.996	1.005	1.092***	.996	1.003	1.001	.982**	.999	1.002	.978**	1.008
FRA	1.085***	.996*	.997	1.095***	.984***	1.007*	.995	.985**	.990	1.011	.990	1.008
NET	1.152***	1.003	1.006	1.145***	.997	1.011**	1.033*	.969***	.999	1.065**	.974*	1.000
NOR	1.099***	.989***	.985***	1.093***	.983***	.991***	1.030***	.968***	.983***	1.020***	.983***	.985**
*East European countries*												
BUL	1.061***	.960***	.976***	1.065***	.961***	.960***	.994	.977***	1.003	.993	.988	1.003
CZE	1.049***	.967***	.993	1.068***	.978***	.996	1.014***	.980***	.995	1.005	.993	.992
EST	1.063***	.975***	.993*	1.072***	.976***	.993	1.030***	.984***	.992	1.005	.972***	1.015*
GEO	1.040***	.986***	.990**	1.050***	.988**	.991*	.991	1.001	1.003	1.009	.987	.995
HUN	1.093***	.968***	.990	1.090***	.965***	.995	1.026***	.966***	1.001	1.008	.987*	.993
LIT	1.054***	.969***	.993	1.069***	.971***	1.008	1.007	.987**	.998	.993	.986**	1.003
POL	1.057***	.977***	1.007	1.075***	.974***	1.008	1.016***	.973***	1.006	1.008	.987**	1.000
ROM	1.058***	.950***	.976*	1.065***	.954***	.951***	.979***	.979***	1.006	.979***	.973***	1.007
RUS	1.030***	.977***	.995	1.020***	.974***	1.004	1.010**	.979***	.995	.986*	.995	1.008

^a ^ Controlled for age, age^2^, and age^3^ (results not shown in the table)

* *p* < .05; ** *p* < .01; *** *p* < .001

For the models comparing the association of the independent variables with the risk to have a first birth while being single versus within marriage for women (see Panel 2 of Tables 3 and 5), we find that including own education in the model reduces the size of the negative coefficient of parental education and in some cases the coefficient even becomes positive. While in the initial models a significant negative association of parental education with single births was found in nine countries, this is reduced to three countries—the US, Austria, and Norway—in the models including own educational attainment.

### Gender Differences: Comparing Births Within Cohabitation Versus Within Marriage

As discussed in the data quality section, there is little reason to expect substantial underreporting of births within cohabitation and marriage for men. This is supported by the descriptive results (Table 2, Panel 1), the changes in the distribution of births to cohabiting men over cohorts resemble the pattern observed for women.

The association of parental education with the risk of having a birth within cohabitation versus within marriage is fairly similar for men and women in most countries. Only in the Czech Republic, Lithuania, and Russia, a negative association is found for women, while for men no significant difference between the higher and lower educated is found, however, note that in the Czech Republic the association almost reaches significance for men (*b* = .985; *p* = .054).

Several gender differences are found regarding the changing association of parental education over cohorts. In Bulgaria, Hungary, and Romania, the negative association of parental education does not significantly change across cohorts for women, while the association becomes more negative for men. In Austria, France, and Estonia, the opposite is found, in these countries the association of parental educational attainment does not change over time for men, while it does change for women (see paragraph 4.3 for the discussion on the result for women).

For the model including a respondent’s own educational attainment, we only find different results for men as compared to women in three countries. In France and the Netherlands, a positive and significant association is found for men and no significant differences are found for women (the *p*-value is .045 for French men). In Estonia, we find a similar negative coefficient of parental education for men and women, however, for men this coefficient does not reach significance, although both *p*-values balance around the .05 level (for women: *p* = .039; for men *p* = .078).

### Gender Differences: Comparing Births While Being Single Versus Within Marriage

The descriptive results (Table 2, Panel 2) show that overall the distribution of single births for men and women follow a similar pattern across cohorts. However, taking all cohorts together, the total percentages of births to singles reveal that in all countries, this percentage is lower for men than for women which hints to a tendency of men to underreport this type of births. In the majority of countries, the differences of the percentage between men and women are rather small. In fact, in 11 countries the differences are 3 percentage points or less. However, in relative terms these differences can still be substantial. We will therefore only discuss the results for the countries for which the underreporting of men is less than 25%^10^ compared to the percentage of women (Canada, the US, Austria, Georgia, and Hungary), and where the results lead to different conclusions for men than for women.

Gender differences in the association of parental education on the risk of having a birth while being single versus within marriage are found in Austria and Canada (Table 3, Panel 2). In these countries, the coefficients for both men and women are negative, but they do not reach statistical significance for men.

Regarding the changes in the association of parental educational attainment over cohort (Table 4, Panel 2), a few gender differences are observed as well. In Hungary, the negative association of parental education does not change significantly over cohorts for women, while it becomes more negative over time for men. In Canada and Austria, only changes in the association of parental education are found for women (see paragraph 4.3), but not for men.

The model that includes own educational attainment (Table 5, Panel 2), shows very similar results for men and women. Only in Austria, gender differences are observed. Here, the association of parental education remains statistically significant after including own educational attainment for women, while no statistical significant association of parental education is found for men in any of the models.

## Discussion

Previous research has found that children growing up with parents with a lower socio-economic status (SES) are more likely to have non-marital births (e.g. Aassve 2003; Wu 1996). Given that children born out of wedlock have poorer life chances, this process contributes to the intergenerational reproduction of inequality. However, most of this research is conducted in the US and only distinguishes between marital and non-marital childbearing. The first research question of this paper was therefore whether parental educational attainment influences the partnership context at first birth similarly across different countries. We thereby distinguished births within cohabitation from births within marriage and births outside a coresidential partnership. The results show that in North American and East European countries, a higher parental education lowers the risk of women to have a birth within cohabitation compared to having a birth within marriage. In contrast, in most West European countries, parental education was not significantly related to the risk of having a birth within cohabitation. In the Netherlands, a positive association of parental education was found. A plausible explanation of this pattern is that in West European countries, which are most advanced in the Second Demographic Transition, births to cohabiting couples have become widely accepted among all social strata. As a result, childbearing within cohabitation is (no longer) more common among the lower socio-economic strata. This is also endorsed by the finding that in Belgium, the Netherlands, and France the predicted association of parental SES is positive for women who grew up in the 1960s and 1970s, a period when the SDT started and cohabitation is assumed to be especially common among higher educated women (Lesthaeghe and Surkyn 2002). In France and the Netherlands, the association becomes less positive with consecutive cohorts. The same was found by Perelli-Harris et al. (2010b) for own educational attainment in France, but they did not find this pattern in the Netherlands. Yet, Norway was the only West European country for which a negative gradient of parental education was found. And precisely this country is often considered to be the most advanced in the transition, which seems to go against the SDT argument. Perhaps, as other scholars have argued, especially in a country like Norway where childbearing within cohabitation is not stigmatized, young couples with lower economic prospects choose this context for childbearing and wait with marriage until they have reached a sufficient level of economic stability (Perelli-Harris et al. 2010b).

In North American countries, and in half of the West and East European countries, we found that women with lower educated parents have a higher risk of having a birth while being single, compared to having a birth within marriage. In North America and four East European countries (Czech Republic, Estonia, Hungary, and Russia), this negative association coincides with a negative association between parental education and the risk of having a birth within cohabitation. Births to cohabiting couples in these East European countries thus appear to be part of a pattern of disadvantage together with births to single mothers, something previous research also observed in the US (Oppenheimer 2003). Single-country studies focusing on own educational attainment already found this in Hungary and Russia (Mikolai 2012; Perelli-Harris and Gerber 2011). Our results show that this conclusion could be extended to the Czech Republic and Estonia. It could thus be that in these countries, due to for example high economic inequality or low social policy expenditure, women with lower status parents face more constraints when growing up, which in turn decreases their risk of having a marital birth. Moreover, in some cases, specific social policies may play a role. Some argue that mothers with limited economic prospects are less likely to decide to live with their partner when welfare benefits are more favourable towards low income, single-headed families (González 2007; Rosenzweig 1999). In Bulgaria, Georgia, Lithuania, and Romania, instead a pattern was found where parental education is negatively related to the risk of having a birth within cohabitation, while it did not significantly influence the risk of having a birth while being single. Possibly the group of single parents in these countries is different in that it includes couples who deliberately choose to live separately from each other and with their family until they have found proper housing and can afford to share a household together (Mikolai 2012).

The second research question was how consistent the association of parental education is across cohorts within these countries. In many countries, the association of parental education in fact changed over birth cohorts. However, no clear pattern was observed. While in some countries a negative association became stronger across cohorts, in other countries a negative gradient became weaker across cohorts or did not change. Moreover, often a change was only found for either one of the partnership contexts (while being single or within cohabitation). There also did not appear to be clear patterns between the different groups of countries. This low level of consistency in the pattern between and within countries complicates the identification of societal changes that can explain the results. We therefore have to leave it to future research to further explore changes over time.

Lastly, we wanted to know to what extent the association of parental education changes when a person’s own educational attainment is included in the model. In most countries, the initial negative coefficient of parental education on the risk of having a first birth within cohabitation or while being single became smaller after including information on respondents’ own educational attainment. For births within cohabitation, the negative associations of parental education, however, remained statistically significant in the North American countries, Norway, and half of the Eastern European countries. For women who had a birth while being single, this was less often the case, as after including own educational attainment, the initial negative association only remained statistically significant in the US, Austria, and Norway. Our expectation was that including own educational attainment in the model could reduce the association of parental education because US research has shown that part of the association between parental SES and family structure can be explained by the intergenerational transmission of SES (Musick and Mare 2006), a mechanism which is proved to exist in other Western societies too (Breen and Jonsson 2005). The results are therefore in line with our expectation. However, our models might underestimate the remaining association of parental education because own education (but not parental education) might suffer from reverse causation, which could inflate the association between own education and union status at childbirth, thereby possibly downsizing the remaining association of parental education. We therefore leave it to future research to study the mediation pathways more thoroughly.

It is important to acknowledge some limitations of the present study. First, parental educational attainment was used as an indicator of parental SES. Although this strategy is often used in the literature, it would be interesting to replicate the analyses using additional indicators of parental SES, such as parental occupational status and income. Second, our analyses were restricted to the role of (parental and respondent’s own) education and cohort. In view of other possible mechanism linking parental SES and partnership at first birth, additional insight into the topic could be gained by including additional childhood indicators (e.g. composition of the childhood family, parental divorce).

Most fertility research has focused on women. One additional objective of this paper was to examine whether the patterns for men and women are comparable across and within countries. In fact, the association of parental education was remarkably similar for men and women. The model including own educational attainment also did not differ much between men and women. This supports the idea that in most instances the mechanisms explaining partnership context at birth are fairly similar for men and women. We therefore recommend to study data of men in addition to that of women more often in fertility research, as it can serve as a test of the robustness of one’s findings in a certain context. In the few cases where statistical gender differences were found, the overall association of parental education was more negative for women than for men. This is in line with previous research suggesting that the mechanism of socialization and role modelling could potentially be stronger for daughters than for sons. Moreover, in quite some countries differences between men and women were found for the interaction model, indicating that the association of parental education changes differently across cohorts for men and women. This could be an interesting topic for further study. It could also be fruitful to expand analyses to higher-order births. In some countries, cohabiting parents are likely to get married after their first birth (Perelli-Harris et al. 2012). If people with higher parental education are more likely to get married after their first birth, while people with lower educated parents are more likely to remain cohabiting or even to separate, the association of parental education might be rather different for higher-order births.

## Footnotes

1 Information was missing on: timing of birth of the respondent (0.5%); partnership situation at first birth (2.3%); respondent’s own educational attainment (0.7%); and parental educational attainment (5.7%). Moreover, 0.2% of the sample was deleted because the first birth occurred before the age of 15.

2 The French data contain information on whether cohabiting couples have registered their union, called Pacte Civil de Solidarité (PACS). We have regarded these couples as cohabiting couples.

3 In the GGS, the questions on father’s and mother’s education were only asked whether the child grew up with at least one of the biological parents. As a consequence, in countries with a high percentage of children who did not grow up with either one of their parents, the number of missings on parental education could be rather high. This was the case for Lithuania and Russia, where the number of missings on parental education exceeds 10%.

4 Some theories suggest that mothers are more important in the socialization of daughters and fathers in socializing their sons (Goslin and Aldous 1969). Using the mean of father’s and mother’s educational attainment might in that case lead to a reduction in the magnitude of the coefficients. Additional analyses—available upon request—show that mother’s (father’s) educational attainment was not consistently more important for women (men) than for men (women).

5 Since the data lack more detailed information on the educational history, the assumption was made that the respondent stayed in school continuously until finishing education.

6 To assure that the final level of education did not become improbably high for respondents still enrolled in school, we assumed an increase until the mean age of reaching the highest educational level in a country or until the highest possible ISLED level in a country.

7 When less than 80% of the respondents in a country indicated when they finished a certain ISLED level, the median age of reaching the corresponding ISCED level over all countries was used instead.

8 As a sensitivity analysis, we ran the same models but included parental educational attainment as a categorical variable, comparing low (ISCED 0–2) with middle (ISCED 3–4) and high (ISCED 5–6) levels. The results strongly resemble the results based on the models including ISLED.

9 We also estimated a model that included school enrolment, which did not lead to large changes in the association between parental education and the outcome variable. Because the quality of the school enrolment variable for the US is questionable and because school enrolment could be endogenous (with partner status at conception influencing school enrolment as married people are less likely to terminate school in the period between conception and birth than cohabiters and singles), we decided to present the model without school enrolment.

10 While the cut-off point of 25% is arbitrary, it errs on the conservative side by omitting countries where the difference between men and women clearly suggests underreporting.

## References

[CR1] Aassve A (2003). The impact of economic resources on premarital childbearing and subsequent marriage among young American women. Demography.

[CR2] Amato PR, Landale NS, Havasevich-Brooks TC, Booth A, Eggebeen DJ, Schoen R (2008). Precursors of young women’s family formation pathways. Journal of Marriage and Family.

[CR3] Axinn WG, Thornton A (1993). Mothers, children, and cohabitation: The intergenerational effects of attitudes and behavior. American Sociological Review.

[CR4] Baizán P, Domínguez M, González MJ (2014). Couple bargaining or socio-economic status? Why some parents spend more time with their children than others. European Societies.

[CR5] Barber JS (2000). Intergenerational influences on the entry into parenthood: Mothers’ preferences for family and nonfamily behavior. Social Forces.

[CR6] Barlow A, Probert R (2004). Regulating marriage and cohabitation: Changing family values and policies in Europe and North America—an introductory critique. Law and Policy.

[CR7] Béchard M, Marchand I (2008). Cycle 20: Family transitions (2006)—Public use microdata file documentation and user’s guide.

[CR8] Bernhardt E, Hoem B (1985). Cohabitation and social background: Trends observed for Swedish women born between 1936 and 1960. European Journal of Population/Revue européenne de Démographie.

[CR9] Berrington A (2001). Entry into parenthood and the outcome of cohabiting partnerships in Britain. Journal of Marriage and Family.

[CR10] Bianchi S, Cohen PN, Raley S, Nomaguchi K, Neckerman KM (2004). Inequality in parental investment in child-rearing: Expenditures, time, and health. Social inequality.

[CR11] Breen R, Jonsson JO (2005). Inequality of opportunity in comparative perspective: Recent research on educational attainment and social mobility. Annual Review of Sociology.

[CR12] Bumpass LL, Raley RK (1995). Redefining single-parent families: Cohabitation and changing family reality. Demography.

[CR13] CBS (2012). Documentatierapport Onderzoekgezinsvorming 2008V1.

[CR14] Cherlin A, Cross-Barnet C, Burton LM, Garrett-Peters R (2008). Promises they can keep: Low-income women’s attitudes toward motherhood, marriage, and divorce. Journal of Marriage and Family.

[CR15] Coleman D, Garssen J (2002). The Netherlands: Paradigm or exception in Western Europe’s demography. Demographic Research.

[CR16] Crettaz E, Jacot C (2014). Do family policies matter for educational outcomes?. European Societies.

[CR17] Edin K (2000). What do low-income single mothers say about marriage?. Social Problems.

[CR18] Edin K, Kefalas M (2005). Promises I can keep: Why poor women put motherhood before marriage.

[CR19] Ermisch J, Wu LL, Wolfe B (2001). Cohabitation and childbearing outside marriage in Britain. Out of wedlock: Causes and consequences of nonmarital fertility.

[CR20] Ermisch J, Francesconi M (2000). Cohabitation in Great Britain: Not for long, but here to stay. Journal of the Royal Statistical Society: Series A (Statistics in Society).

[CR21] Eurostat (2006). Population statistics.

[CR22] Eurostat (2015). European social statistics.

[CR23] Finer LB, Zolna MR (2014). Shifts in intended and unintended pregnancies in the United States, 2001–2008. American Journal of Public Health.

[CR24] Fokkema T, Kveder A, Hiekel N, Emery T, Liefbroer AC (2016). Generations and Gender Programme Wave 1 data collection: An overview and assessment of sampling and fieldwork methods, weighting procedures, and cross-sectional representativeness. Demographic Research.

[CR25] Forrest JD (1994). Epidemiology of unintended pregnancy and contraceptive use. American Journal of Obstetrics and Gynecology.

[CR26] Forste R, Jarvis J (2007). “Just like his dad”: Family background and residency with children among young adult fathers. Fathering.

[CR27] Goldstein, J. R., & Kenney, C. T. (2001). Marriage delayed or marriage forgone? New cohort forecasts of first marriage for US women. *American Sociological Review*, *66*(4), 506–519.

[CR28] González L (2007). The effect of benefits on single motherhood in Europe. Labour Economics.

[CR29] Goslin DA, Aldous J (1969). Handbook of socialization theory and research.

[CR30] Haveman R, Wolfe B (1995). The determinants of children’s attainments: A review of methods and findings. Journal of economic literature.

[CR31] Heard G (2011). Socioeconomic marriage differentials in Australia and New Zealand. Population and Development Review.

[CR32] Heuveline P, Timberlake JM (2004). The role of cohabitation in family formation: The United States in comparative perspective. Journal of Marriage and Family.

[CR33] Heuveline P, Timberlake JM, Furstenberg FF (2003). Shifting childrearing to single mothers: Results from 17 western countries. Population and Development Review.

[CR34] Hiekel N, Castro-Martín T (2014). Grasping the diversity of cohabitation: Fertility intentions among cohabiters across Europe. Journal of Marriage and Family.

[CR35] Hoem JM, Kreyenfeld M (2006). Anticipatory analysis and its alternatives in life-course research Part 1: Education and first childbearing. Demographic Research.

[CR36] Hofferth SL, Goldscheider F (2010). Family structure and the transition to early parenthood. Demography.

[CR37] Hoffman, S. D., Foster, E. M., & Furstenberg Jr, F. F. (1993). Reevaluating the costs of teenage childbearing. *Demography*, *30*(1), 1–13. 8379973

[CR38] Högnäs RS, Carlson MJ (2012). “Like parent, like child?”: The intergenerational transmission of nonmarital childbearing. Social Science Research.

[CR39] Högnäs RS, Thomas JR (2016). Birds of a feather have babies together? Family structure homogamy and union stability among cohabiting parents. Journal of Family Issues.

[CR40] Joyner K, Peters HE, Hynes K, Sikora A, Taber JR, Rendall MS (2012). The quality of male fertility data in major US surveys. Demography.

[CR41] Kalmijn M (2011). The influence of men’s income and employment on marriage and cohabitation: Testing Oppenheimer’s theory in Europe. European Journal of Population/Revue européenne de Démographie.

[CR42] Kennedy S, Bumpass L (2008). Cohabitation and children’s living arrangements: New estimates from the United States. Demographic Research.

[CR43] Kiernan K (2001). The rise of cohabitation and childbearing outside marriage in Western Europe. International Journal of Law, Policy and the Family.

[CR44] Kiernan K, Moynihan DP, Smeeding T, Rainwater L (2004). Unmarried cohabitation and parenthood: Here to stay? European perspectives. The future of the family.

[CR45] Kreyenfeld M, Hornung A, Kubisch K, Jaschinski I (2010). Fertility and union histories from German GGS data: some critical reflections (Vol. 23, Working paper).

[CR46] Lesthaeghe R (2010). The unfolding story of the second demographic transition. Population and Development Review.

[CR47] Lesthaeghe, R., & Surkyn, J. (1988). Cultural dynamics and economic theories of fertility change. *Population and Development Review*, *14*(1), 1–45.

[CR48] Lesthaeghe R, Surkyn J (2002). New forms of household formation in Central and Eastern Europe: Are they related to newly emerging value orientations?.

[CR49] Levels M, Sluiter R, Need A (2014). A review of abortion laws in Western-European countries. A cross-national comparison of legal developments between 1960 and 2010. Health Policy.

[CR50] Liefbroer AC, Elzinga CH (2012). Intergenerational transmission of behavioural patterns: How similar are parents’ and children’s demographic trajectories?. Advances in Life Course Research.

[CR51] Martin, J. A., Hamilton, B. E., Osterman, M. J., Curtin, S. C., & Mathews, T. J. (2015). *Births: Final data for 2013. national vital statistics reports: from the centers for disease control and prevention* (Vol. 64). National Center for Health Statistics: National Vital Statistics System.

[CR52] McLanahan S (2009). Fragile families and the reproduction of poverty. The Annals of the American Academy of Political and Social Science.

[CR53] McLanahan S, Sandefur G (1994). Growing up with a single parent: What helps, what hurts.

[CR54] Mikolai J (2012). With or without you: Partnership context of first conceptions and births in Hungary. Demográfia. English Edition.

[CR55] Miller BC (2002). Family influences on adolescent sexual and contraceptive behavior. Journal of sex research.

[CR56] Musick K (2002). Planned and unplanned childbearing among unmarried women. Journal of Marriage and Family.

[CR57] Musick K, Mare RD (2004). Family structure, intergenerational mobility, and the reproduction of poverty: Evidence for increasing polarization?. Demography.

[CR58] Musick K, Mare RD (2006). Recent trends in the inheritance of poverty and family structure. Social Science Research.

[CR59] OECD (2011). Divided we stand: Why inequality keeps rising.

[CR60] OECD (2015). Economic policy reforms 2015: Going for growth.

[CR61] Oppenheimer VK (2003). Cohabiting and marriage during young men’s career-development process. Demography.

[CR62] Oppenheimer VK, Kalmijn M, Lim N (1997). Men’s career development and marriage timing during a period of rising inequality. Demography.

[CR63] Perelli-Harris B, Gassen NS (2012). How similar are cohabitation and marriage? Legal approaches to cohabitation across Western Europe. Population and Development Review.

[CR64] Perelli-Harris B, Gerber TP (2011). Nonmarital childbearing in Russia: Second demographic transition or pattern of disadvantage?. Demography.

[CR65] Perelli-Harris B, Kreyenfeld M, Kubisch K (2010). Technical manual for the harmonized histories database (Vol. 11, MPIDR Working paper).

[CR66] Perelli-Harris B, Kreyenfeld M, Sigle-Rushton W, Keizer R, Lappegård T, Jasilioniene A (2012). Changes in union status during the transition to parenthood in eleven European countries, 1970s to early 2000s. Population Studies.

[CR67] Perelli-Harris B, Sigle-Rushton W, Kreyenfeld M, Lappegard T, Keizer R, Berghammer C (2010). The educational gradient of childbearing within cohabitation in Europe. Population and Development Review.

[CR68] Raffaelli M, Ontai LL (2004). Gender socialization in Latino/a families: Results from two retrospective studies. Sex Roles.

[CR69] Rendall MS, Clarke L, Peters HE, Ranjit N, Verropoulou G (1999). Incomplete reporting of men’s fertility in the United States and Britain: A research note. Demography.

[CR70] Rindfuss, R. R., & VandenHeuvel, A. (1990). Cohabitation: A precursor to marriage or an alternative to being single? *Population and Development Review*, *16*(4), 703–726.

[CR71] Rosenzweig MR (1999). Welfare, marital prospects, and nonmarital childbearing. Journal of Political Economy.

[CR72] Rowlingson K, McKay S (2005). Lone motherhood and socio-economic disadvantage: Insights from quantitative and qualitative evidence. The Sociological Review.

[CR73] Schröder H (2014). Levels and loadings—Two methods to improve the measurement of education in comparative research.

[CR74] Schröder H, Ganzeboom HB (2014). Measuring and modeling level of education in European societies. European Sociological Review.

[CR75] Shattuck, R. (2015). *Does it matter if she’d rather marry? The role of individual preferences in young women’s likelihood of a nonmarital first birth.* (Working paper): Maryland Population Center.

[CR76] Thomson E, McLanahan SS, Curtin RB (1992). Family structure, gender, and parental socialization. Journal of Marriage and Family.

[CR77] Thornton A, Camburn D (1987). The influence of the family on premarital sexual attitudes and behavior. Demography.

[CR78] van de Kaa DJ (2001). Postmodern fertility preferences: From changing value orientation to new behavior. Population and Development Review.

[CR79] Vergauwen J, Wood J, De Wachter D, Neels K (2015). Quality of demographic data in GGS Wave 1. Demographic Research.

[CR80] Wu LL (1996). Effects of family instability, income, and income instability on the risk of a premarital birth. American Sociological Review.

[CR81] Wu LL, Martinson BC (1993). Family structure and the risk of a premarital birth. American Sociological Review.

